# Perspectives on the underlying drivers of urgent and emergency care reconfiguration in Ireland

**DOI:** 10.1002/hpm.2469

**Published:** 2017-10-26

**Authors:** E. Droog, C. Foley, O. Healy, C. Buckley, M. Boyce, S. McHugh, J.P. Browne

**Affiliations:** ^1^ Department of Epidemiology and Public Health, Faculty of Medicine and Health University College Cork Cork Ireland; ^2^ Department of Public Health, HSE South Region St. Finbarr's Hospital Cork Ireland

**Keywords:** evidence, finance, politics, stakeholder perspectives of change, urgent and emergency care reconfiguration

## Abstract

**Background:**

There is an increasing tendency to reconfigure acute hospital care towards a more centralised and specialised model, particularly for complex care conditions. Although centralisation is presented as “evidence‐based”, the relevant studies are often challenged by groups which hold perspectives and values beyond those implicit in the literature. This study investigated stakeholder perspectives on the rationale for the reconfiguration of urgent and emergency care in Ireland. Specifically, it considered the hypothesis that individuals from different stakeholder groups would endorse different positions in relation to the motivation for, and goals of, reconfiguration.

**Methods:**

Documentary analysis of policy documents was used to identify official justifications for change. Semi‐structured interviews with 175 purposively sampled stakeholders explored their perspectives on the rationale for reconfiguration.

**Results:**

While there was some within‐group variation, internal and external stakeholders generally vocalised different lines of argument. Clinicians and management in the internal stakeholder group proposed arguments in favour of reconfiguration based on efficiency and safety claims. External stakeholders, including hospital campaigners and local political representatives expressed arguments that focused on access to care. A “voter” argument, focused on the role of local politicians in determining the outcome of reconfiguration planning, was mentioned by both internal and external stakeholders, often in a critical fashion.

**Conclusion:**

Our study adds to an emerging literature on the interaction between a technocratic approach to health system planning advocated by clinicians and health service managers, and the experiential “non‐expert” claims of the public and patients.

## 1 INTRODUCTION

Health care reconfiguration has been defined as a “…deliberately induced change of some significance in the distribution of medical, surgical, diagnostic and ancillary specialties that are available in each hospital or other secondary or tertiary acute care unit in locality, region or healthcare administrative area”.^1^ Within the acute hospital sector, there is an increasing tendency to reconfigure towards a more centralised and specialised model, particularly for complex care conditions.^2^ This is characterised by the consolidation of services across a region on fewer hospital sites serving a higher volume of patients, to improve efficiency and outcomes.^3^ Several studies have supported the benefits of high volume, specialised units for coronary, stroke, and major trauma patients because of an apparent association with better outcomes.^4^, ^5^, ^6^, ^7^

### 1.1 Perspectives on the evidence

Although centralisation is presented as “evidence‐based”, the relevant studies are often challenged by stakeholders who hold perspectives and values beyond those implicit in the literature.^8^ Proposals to centralise services often face public opposition, arising from concerns about future access to services.^9^, ^10^ Plans to downgrade or close Emergency Departments have been particularly controversial.^2^ It has been argued that volume should not be understood in isolation as a justification for centralising emergency care, particularly as the volume‐outcome relationship has not been studied for many emergent conditions where fast access is important. It has also been argued that other factors such as geography and population needs may support a less centralised model in many contexts.^11^ Centralising care can have unexpected impacts on sub‐groups of the population.^3^ Some groups, such as the elderly, those with low socioeconomic status, and those with suboptimal transport access, may reduce their use of health care services when journey distances increase due to the increased costs associated with added travel.^12^, ^13^ Also, increased journey distances may result in an increased risk of mortality for some patients, offsetting any benefits that may arise from receiving care at a specialist unit.^14^

Public sector health care bodies have been characterised as “pluralistic” in the organisational literature^15^and should, ideally, satisfy the requirements of actors within and outside of the organisation in order to garner their support.^8^, ^16^ It has been suggested that policymakers have tended to prioritise efficiency and effectiveness criteria when configuring health care, rather than seeking to balance the priorities of different stakeholders and fitting a solution to incorporate these priorities.^15^, ^17^ This implies that implementing “out of the box” reconfiguration based on international evidence on centralisation alone is not likely to be accepted by stakeholders in different contexts.

### 1.2 Framework of perspectives on reconfiguration

**Figure 1:**
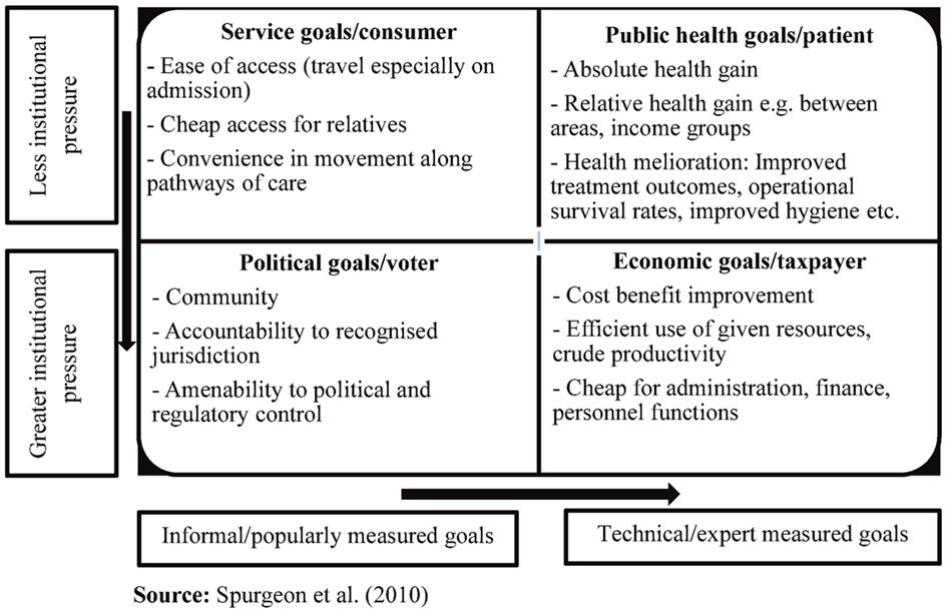
Classification for types of goals for health care system reconfiguration [Colour figure can be viewed at http://wileyonlinelibrary.com]

Spurgeon et al^10^ synthesised previous research on perspectives on reconfiguration, particularly centralisation,^2^, ^18^ into a framework of 4 arguments. Those in favour of reconfiguration typically employ an economic “taxpayer” argument about efficiency and cost‐benefit improvement and a public health “patient” argument based on the likelihood of improved outcomes. Those against put forward a “consumer” argument based on a demand for easy, convenient, and affordable access, and a politically driven “voter” argument based on a demand for local control of services. The framework distinguishes between “popular” and “technical” goals and highlights the role of “institutional pressures”, such as financial constraints and regulatory initiatives, in shaping the goals of different stakeholders. In this study, we use the framework (Figure 1) to investigate perspectives on the underlying drivers of urgent and emergency care reconfiguration in the Republic of Ireland. Specifically, we investigate the extent to which individuals from different stakeholder groups endorse different positions in relation to the motivation for, and goals of, reconfiguration.

### 1.3 Context

The Health Service Executive (HSE) is the public sector body responsible for the delivery of urgent and emergency care in Ireland through emergency departments, acute assessment units, and minor injury units. In 2006, the HSE introduced a “Transformation Programme” designed to change the organisation of care delivery across 8 regional hospital networks.^19^ Urgent and emergency care was a specific service reconfiguration project and favoured a “hub and spoke” model. The “hubs” were larger hospitals where complex care was proposed to be centralised. The “spokes” were smaller hospitals which were to focus on lower severity cases. The reconfiguration initiatives occurred during a period when the Irish health care regulator was carrying out investigations into the quality of care provided by a small number of low volume Emergency Departments.^20^, ^21^ These investigations prompted a broader HSE review of small Emergency Departments and provided further impetus to the reconfiguration programme. To date, the implementation of this programme has differed across the country. Regional characteristics and a summary of the reconfiguration undertaken are shown in Table 1. The changes have largely consisted of closing or “re‐designating” (ie, downgrading) the function of smaller Emergency Departments and associated acute services in the Southern, Western, Mid‐Western, and North‐Eastern parts of the country, and centralising complex care to larger tertiary hospitals in Dublin, Cork, Galway, and Limerick. Campaigns to resist these changes were organised in all 4 regions. Similar changes were planned for the South‐Eastern region but were abandoned. There has been little attempt to reconfigure urgent and emergency care in Dublin city although some rationalisation of individual services such as coronary and stroke care has occurred and 1 small Emergency Department close to the city was changed to a local injury unit. The country now has radically different models of provision in different regions, and these variations do not seem to be related to underlying geographical constraints. The South‐Eastern region, for example, currently has 4 Emergency Departments and a population of 511 070 (1 per 127 767 persons), while the Mid‐Western region has 1 Emergency Department for a population of 385 172.^22^ These regions have very similar geographies and population densities. County Dublin, which is largely urban, has 6 Emergency Departments and a population of 1 345 402 (1 per 224 233 persons).^22^

**Table 1:** Regional characteristics and summary of reconfiguration undertaken

Significant reconfiguration		
Region	Characteristics	Summary of regional change
**South** (Cork and Kerry)	Population: 689 750 Area (km^2^): 12 161	**Regional reconfiguration** • region‐specific reconfiguration plan largely implemented, beginning 2012–2013 • region‐wide clinical governance structures established • single general practice (GP) out of hours co‐operative **Emergency department services reconfiguration** • acute stroke, coronary and major trauma care provided at hub in Cork [Cork University Hospital] with support of ambulance protocols and outlying centres [Kerry: University Hospital Kerry; Cork: Bantry General Hospital] • 2 EDs reconfigured to local injury units [Cork: Mallow General Hospital (2013) and Bantry General Hospital (2013)] • one emergency department (ED) closed [Cork: South Infirmary Hospital (2012)]
**Mid‐west** (Limerick, Clare and Tipperary North)	Population: 385 172 Area (km^2^): 8252	**Regional reconfiguration** • region‐specific reconfiguration plan largely implemented, 2009–2013 • ambulance bypass protocols and region‐wide clinical directorates established • single GP out of hours co‐operative **Emergency department services reconfiguration** • all emergency care centralised to 1 hospital [Limerick: University Hospital Limerick] • 2 EDs reconfigured to local injury units [Clare: Ennis Hospital (2009); Tipperary North: Nenagh Hospital (2009)
***Some reconfiguration***		
**Region**	**Characteristics**	**Summary of regional change**
**West** (Galway, Roscommon, Mayo, Leitrim, Sligo, Donegal)	Population: 709 497 Area (km^2^): 22 649	**Regional reconfiguration** • clinical directorates established across the region • several out of hours GP co‐operatives **Emergency department services reconfiguration** • single hub for acute coronary and major trauma care [Galway: University Hospital Galway] with major trauma support services provided at other centres [Mayo: Mayo University Hospital; Donegal: Letterkenny University Hospital; Sligo: Sligo University Hospital]. Acute stroke care at all centres, excluding Roscommon general hospital • 1 ED reconfigured to local injury unit [Roscommon: Roscommon General Hospital (2011)] • no ED in Leitrim
**North‐east** (Cavan, Meath, Louth and Monaghan)	Population: 460 682 Area (km^2^): 6395	**Regional reconfiguration** • region‐specific reconfiguration plan partly implemented from 2006 to 2010 • limited regional clinical governance • roll‐out of general practitioner (GP) out of hours care **Emergency department services reconfiguration** • some centralisation of trauma, acute stroke and coronary care [Cavan: Cavan General Hospital; Louth: Our Lady of Lourdes Drogheda] with rehab support in other hospitals • Dublin North [Mater Hospital] is the percutaneous coronary intervention (PCI) centre with supporting ambulance protocols • 2 emergency departments reconfigured to local injury units [Louth: Louth County Hospital (2010); Monaghan: Monaghan General Hospital (2009)]
**South‐east** (Carlow, Kilkenny, Wexford, Waterford and Tipperary south)	Population: 511 070 Area (km^2^): 9451	**Regional reconfiguration** • informal clinical network with shared regional rota for emergency medicine consultants • single GP out of hours co‐operative. **Emergency department services reconfiguration** • designated hub for major trauma, and acute coronary care [Waterford: Waterford Regional Hospital—PCI Centre supported out of hours by Cork] with ambulance bypass protocols • acute stroke care available at all 4 hospitals • no ED in Carlow
**Dublin south** (Dublin South City, Dun Laoghaire Rathdown, Wicklow)	Population: 590 952 Area (km^2^): 2168	**Regional reconfiguration** • multiple out of hours GP co‐operatives **Emergency department services reconfiguration** • centralisation of acute stroke, coronary, and trauma care to 2 hospitals (both in Dublin South City) but limited differentiation and integration between both • 1 ED reconfigured to local injury unit [Dun Laoghaire Rathdown: St Columcille's Hospital (2013)] • 1 ED with reduced hours [Dun Laoghaire Rathdown: St Michaels (2003)] • no ED in Wicklow
***Little reconfiguration***		
**Region**	**Characteristics**	**Summary of regional change**
**Dublin north‐east** (Fingal, Dublin North City)	Population: 618 033 Area (km^2^): 532	**Regional reconfiguration** • no major changes • out of hours GP co‐operative established **Emergency department services reconfiguration** • 3 large emergency departments with limited governance integration and differentiation of services. PCI Centre established [Dublin North: Mater Hospital]
**Dublin midlands** (Dublin south, Longford, Westmeath, Laois, Offaly, Kildare)	Population: 792 820 Area (km^2^): 8442	**Regional reconfiguration** • limited integration of clinical governance • several out of hours GP co‐operatives operating **Emergency department services reconfiguration** • centralisation of acute stroke [Kildare: Naas General Hospital; Westmeath: Midlands Regional Hospital Mullingar, and Dublin South: Tallaght Hospital) coronary care [Dublin South: Tallaght Hospital] and trauma [Offaly: Midland Regional Hospital Tullamore; Dublin South: Tallaght Hospital) at several hospitals, supported by ambulance bypass protocols • no ED in Longford

(Regional characteristics were referenced from the Central Statistics Office, 2016).

## 2 METHODS

### 2.1 Design and data collection methods

This is a qualitative study using documentary analysis, and interviews with stakeholders across all 8 hospital network regions in Ireland. Documentary analysis was used to identify official justifications for reconfiguration. Document selection was guided by a broad definition of policy to include any published decision or action a Government makes (or chooses not to make) in relation to services,^23^ or a political process consisting of several connected decisions.^24^ Official health policy documents published by the Irish Government or the HSE from 2005 to 2015, and all documents from official state agencies or interest groups that influenced health policy during that period, were reviewed. Draft and unpublished versions were excluded. The websites of the Irish Department of Health, HSE, professional institutes and the Irish national health care regulator were searched, along with reference lists of policy documents obtained. The relevance of all documents selected was discussed and agreed by consensus among the research team. A breakdown of policy documents analysed by domain can be accessed using the link to electronic supplementary material provided at the end of this article.

Semi‐structured interviews were conducted in 2014 and 2015 by 2 interviewers with a range of stakeholders to explore their perspectives on the drivers of urgent and emergency care reconfiguration in Ireland. Participants were sampled using purposive and snowball sampling methods. An initial purposive sample of participants who were centrally involved in the reconfiguration process was identified in policy documents. Snowball sampling was then used to identify a broader group of internal stakeholders, by asking participants to suggest individuals they believed could contribute to the study. External stakeholders were identified by local media coverage of reconfiguration or were suggested by other interviewees.

Stakeholders were categorised according to their role in the health system reconfiguration. The term “internal stakeholders” referred to a heterogeneous group of non‐clinical (management and patient advocates) and clinical (doctors and nurses) staff working in the public health service. It included those directly working within the regional HSE management structures and involved in reconfiguration planning and implementation at either a national or regional level, as well as front‐line clinicians (including clinical leaders with a national remit) working in HSE hospitals. The term “external stakeholders” referred to those working outside the HSE and included public and private ambulance representatives, general practitioners (GPs), private hospital representatives, representatives of civic groups such as campaigns to prevent the closure of local Emergency Departments, local media representatives, and local politicians.

Potential participants were contacted via e‐mail or telephone and invited to participate. Interviews were carried out in 2014 and 2015 in either the participants' or researchers' place of work. The topic guide was based on the framework shown in Figure 1 and can be accessed using the link to electronic supplementary material provided at the end of this article. Recruitment was concluded in each region when the required diversity of stakeholders had been reached. A digital recorder was used for each interview. In addition, the researchers wrote a reflective summary after each interview and held regular debriefing sessions to discuss the memos and to critically examine any potential research bias.

### 2.2 Ethical approval

This study was approved by 2 research ethics committees. All stakeholders were ensured anonymity in the publication of study findings, identified only by region, and provided written consent to participate.

### 2.3 Analysis

Interviews were transcribed by 2 researchers, and emergent findings were discussed regularly with the larger research team. It was agreed that saturation had likely been reached in the first 123 interviews and that full transcription of the remaining interviews was not necessary. The researchers listened to the remaining 52 interviews to ensure that this was the case and selectively transcribed material to provide additional quotes for the themes that had been established in the first 123 interviews. Documents and interviews were imported to NVivo software for data management. Two researchers independently coded half of the interview data each. To ensure consistency in application of the coding framework, the researchers met regularly to compare coding within and across both data sets and discuss if additional codes should be added. A framework analysis approach was used for both sources of data.^25^ Following familiarisation, policy goals and stakeholder opinions on the reasons for change were initially mapped onto the framework of arguments shown in Figure 1. The framework was initially tested by indexing a sample of the data. This was reviewed by the team before the entire dataset was indexed. Personal memos were used to track decisions and challenge any personal or professional biases in interpreting the data. Findings were refined after discussion with the full research team.

We considered the hypothesis that individuals from different stakeholder groups would endorse different positions in relation to the motivation for, and goals of, reconfiguration. Participants' accounts were classified according to their compatibility with taxpayer, patient, consumer, and voter type interests. Where appropriate, participants' accounts were classified in more than 1 argument of interest. Quotations were selected to represent the essence of each argument.

## 3 RESULTS

Twenty‐two policy documents were analysed. They included official documents relating to all or part of the provision of urgent and emergency care, including national and regional hospital reconfiguration reports, health care regulatory reports at specific hospital sites, and national clinical programme reports. A description of interviewees by region and stakeholder type is shown in Table 2.

**Table 2:** Interviewees by stakeholder position and hospital network region

Region									
Stakeholder position	North‐east (NE)	Dublin‐ north‐east (DNE)	Dublin‐south (DS)	Dublin‐midlands (D‐mid)	South‐east (SE)	South (S)	Mid‐west (MW)	West/ north‐west (W/NW)	Total
**Internal**	**8**	**12**	**11**	**14**	**13**	**20**	**13**	**14**	**105**
Management	3	2	3	3	3	10	4	4	32
Doctor	3	5	5	5	6	6	4	6	40
Nurse	2	3	3	5	4	3	4	3	27
Patient advocate	0	2	0	1	0	1	1	1	6
**External**	**9**	**4**	**8**	**10**	**6**	**16**	**8**	**9**	**70**
Ambulance	0	0	2	3	0	3	2	2	12
GP	3	1	3	3	2	5	2	2	21
Private emergency department	0	1	1	0	0	0	0	1	3
Civic groups	2	1	1	2	0	4	1	1	12
Local media	1	1	0	0	2	2	1	0	7
Local politician	3	0	1	2	2	2	2	3	15
**Total**	**17**	**16**	**19**	**24**	**19**	**36**	**21**	**23**	**175**

The 4 types of argument illustrated in Figure 1 were found to be sufficient to cover the material collected during the study. No regional differences were found, but differences were clearly found across stakeholder groups. A small number of contrarian views within stakeholder groups were present, particularly among internal clinical stakeholders.

### 3.1 “Taxpayer” argument

The “taxpayer” argument for health care reform was obvious in many national^26^, ^27^, ^28^, ^29^, ^30^, ^31^, ^32^ and regional^33^, ^34^, ^35^ policy documents. This argument focused on the efficient use of resources in order to maximise the benefits of public spending. Reorganising care “…in the appropriate location, with reduced duplication and maximum sharing of resources”^28^ was considered optimal in “…getting best value from health system resources”,^26^ and thereby deemed “…efficient for the taxpayer”.^27^ Regional documents echoed this national claim, arguing the need to support the efficient achievement of reconfiguration: “…developing a health service which is efficient and cost effective”.^33^

Many internal (clinical and non‐clinical) stakeholders across the 8 regions used a “taxpayer” style of economic argument to justify their support for reconfiguration. It was claimed that maintaining and developing the current number of acute hospitals at a desirable standard was unaffordable and that not all Emergency Departments were sustainable without increased resourcing for inter‐reliant services such as acute surgery, anaesthesia, and intensive care. These stakeholders also argued that not every Emergency Department attendance was an emergency and that a more efficient streamlining of patients to more appropriate settings was considered a priority: *“… people realised that, right, if we want to keep giving a high standard of care with less resources, we need to do it differently and I think that really was a positive part of the recession. It gave us all a wakeup call and made us realise that we can do things well and we don't need to be spending a fortune”* (Internal nursing stakeholder, Region W/NW). Almost all internal stakeholders across the 8 regions supported the idea of centralisation. This view was also shared by some external stakeholders, mainly GPs, local politicians, and local media, who emphasised the importance of being pragmatic if the service was to be sustainable: *“…an A&E on the corner of every town in every village in the country… just not feasible”* (Politician, Region NE).

However, the “taxpayer” argument was challenged by some internal clinical and external stakeholders who saw reconfiguration as a cost‐saving initiative: *“The driver is always money… It is not patient quality”* (Internal nursing stakeholder, Region DS); *“...they're saving money but they're not saving lives unfortunately. It's the patient who is suffering”* (Hospital campaigner, Region NE). There was also scepticism among some internal clinical and external stakeholders about the claim that centralisation of care led to efficiency gains. Many cited disruptions to safe and efficient care processes at hub Emergency Departments due to an increased volume of patients. These were apparent in increased ambulance handover times, Emergency Department waiting times, and increases in the number of patients on trolleys awaiting admission. The extra costs incurred in dealing with these disruptions, such as hiring locum staff and paying for private ambulance transfers for step‐down care in order to free up acute beds, were referred to as *“…crippling”* the acute hospital service (Internal managerial stakeholder, Region MW). Many internal and external stakeholders questioned the role of smaller hospitals in the aftermath of reconfiguration, claiming that they were being underutilised for elective activity. Some internal stakeholders referred to a culture of bureaucracy underpinning change that creates further inefficiencies: *“They restructure and reconfigure and it just means another layer but they never take out the previous layers”* (Internal nursing stakeholder, Region SE). A concern among several GPs was that their service was increasingly expected to absorb urgent care that had previously been delivered in the hospital setting, without commensurate resources: *“…a cheap commodity and a vehicle for [policy makers] to get what they want for cheaper and deliver a great political promise”* (GP, Region MW).

### 3.2 “Patient” argument

A “patient” argument for reconfiguration, citing the need for quality and safety reforms to improve patient outcomes, was present in many national policy documents.^26^, ^27^, ^28^ A series of regional hospital reconfiguration reports similarly stipulated the need for a critical mass of medical workforce and workload for the purpose of providing a guaranteed quality of acute care.^33^, ^34^, ^35^ Quality and safety arguments were also used to make the case for designing reconfiguration plans around academic partnerships between acute hospitals and designated medical schools.^28^, ^33^

Almost all internal (clinical and non‐clinical) stakeholders used the “patient” argument to justify reconfiguration. There were a number of allusions to international evidence to support the centralisation of health care: *“…international evidence…would suggest that all of these patients do better where they're brought to a site that has a number of different specialties on that site, they can effectively deal with whatever's presented to them”* (Internal managerial stakeholder, Region D‐Mid). Several internal stakeholders referred to investigations by the Irish national health care regulator: *“There had been a number of adverse incidents throughout the country and I think there was a realisation that, you know, we have to improve the quality of care”* (Internal nursing stakeholder, Region W/NW). Concerns raised by the Irish health care regulator about the variability of out‐of‐hours' senior medical cover in small Emergency Departments were described by 1 internal managerial stakeholder as *“…the sole reason”* for reconfiguration (Region NE). Several internal stakeholders stated that smaller Emergency Departments did not have sufficient on‐site support from other acute services such as surgery, anaesthesia and intensive care: *“…if you have no surgeon in a place well then you're not going to have an anaesthetist, are you? If you don't have an anaesthetist you're not going to have an intensive care. If you don't have an intensive care, anaesthetist and a surgeon, you can't really look after trauma, can you?”* (Internal medical stakeholder, Region S). Smaller hospitals were seen by a number of internal stakeholders as requiring formal academic partnerships, as this would increase the number of clinicians rotating through these sites while training, and also increase the interest and involvement of senior clinicians working at hub university hospitals.

Some internal clinical stakeholders were less inclined to agree with broad generalisations about the safety of smaller Emergency Departments. They argued that regulatory investigations at a small number of hospitals had been used to enact a larger wave of changes across the country without evaluating each hospital on a case‐by‐case basis. They felt that planning had become a *“…knee jerk response”* to regulatory pressure (Internal managerial stakeholder, Region DS). Another concern was that insufficient consideration had been given to the post‐reconfiguration volume of patients attending hub hospitals, which had now become *“…a very unpleasant environment in which to experience your healthcare”* (Internal medical stakeholder, Region MW). It was further claimed that the closure of smaller Emergency Departments would inevitably disrupt historical relationships between the smaller local hospital, primary care, and the community: *“…previously I would have known the sister and the junior sister in the A&E and I would know that I could always ring up and most times there was one of them on… I don't know who's who now”* (GP, Region S). There was also a belief that longer journey times would outweigh the benefits of travelling to larger, specialist centres, as well as concerns about the quality of care at hub hospitals due to overcrowding. GPs expressed mixed opinions on the centralised model of care and its impact on the patient. One GP explicitly supported a technical patient safety argument: *“Doing things well you have to be reaching a certain threshold…familiarity breeds good practice”* (Region D‐Mid). Others were concerned that out‐of‐hours primary care would not be able to compensate for the closure of a local Emergency Department and that this would create a separate unanticipated set of safety issues.

Some stakeholders, both internal and external, expressed cynicism about the role of academic clinicians in shaping the outcome of reconfiguration planning and questioned whether academic partnerships were necessary to deliver safe services. It was stated that some reconfiguration plans were biased towards the training and research needs of medical schools rather than local population needs and had disrupted historical relationships between hospitals: *“If it wasn't for the medical schools, the logical thing to do was have the whole North East together”* (Internal medical stakeholder, Region NE). Some stakeholders considered this to be a *“...land grab”* opportunity (Internal medical stakeholder, Region SE) for the sake of building academic partnerships: *“…all that kind of gaming goes on”* (Internal managerial stakeholder, Region SE). It was argued that matching service plans to local needs should trump academia in the order of priority: *“…it's about providing clinical services in the first instance, it's about training in the second instance and then the academics and all these other things … should be the secondary aim or tertiary aim”* (Internal medical stakeholder, Region NE).

### 3.3 “Consumer” argument

Both policy documents and stakeholder accounts referenced the need for an accessible urgent and emergency care system. What this meant and how it should be achieved in the delivery of urgent and emergency care differed among stakeholders. Many policy documents referenced the need to deliver “…enhanced local care provision”,^28^so that patients would “...not have to travel to the larger hospital”.^27^ The notion of balancing the desire for efficiency with the needs of a local community was also recognised: “…developing a health service which is efficient and cost effective but which also is responsive to the needs of communities”.^33^ National clinical programme reports emphasised the need to recognise “… the essential role of large and small hospitals, general practitioners (GPs) and community services” in improving patient care.^31^

Internal stakeholders reported they were aware that access was an important dimension of urgent and emergency care and sought to frame their position as focused on delivering as much care locally as possible while concentrating only complex care in larger specialist centres: *“…we [local rural hospital]… would retain 90 per cent of the activity… Actually we have retained 95 per cent of it to be honest”* (Internal medical stakeholder, Region S). Nevertheless, the implication of a number of statements made by internal stakeholders was that travel distance was considered secondary when formulating reconfiguration plans for patients with complex care needs. Campaigners were often portrayed by internal stakeholders and some GPs as naive in their vision of what was best for the patient: *“I think the public wants a hospital next door. They don't want to travel. They want their hospital right next to them and if you ask the public that's what they'll tell you, but whether that's good for them or not… I think you need to go with the expert opinion on it”* (GP, Region DS).

External stakeholders (mainly hospital campaigners including local political representatives) typically viewed local access to emergency care services as a self‐evident “good” and were willing to provide justification when prompted. They cited the additional risks and financial burden associated with longer travel times: *“[An] emergency occurring in Castletownbere that has to be transported to Cork, you are looking at an absolute minimum, even under blue light, of two and a half hours in ambulance”* (Hospital campaigner, Region S). They also made reference to the importance of familiarity with local services and staff as an often neglected element of high quality care: *“I mean of course we want centres of excellence where people are building up their experience, where you do have the best technology and so on and so forth but that shouldn't mean [taking out] the sort of local point of contact and you know that place which is easy to go to where you're familiar with people”* (Politician, Region DS). Moreover, they expressed satisfaction with the quality of services provided in their local area and believed that smaller hospitals could deliver care that was just as good as larger hospitals if they were properly resourced: *“…a hospital like Monaghan can do just as good if it is resourced properly”* (Hospital campaigner, Region NE). Some external stakeholders asserted that hub hospitals were inferior in some respects because of the overcrowding that had been induced by the closure of smaller Emergency Departments: *“We're just finding that the bigger hospitals are being overloaded”* (GP, Region NE).

### 3.4 “Voter” argument

“Voter” arguments focused on the symbolic and financial value of health care services to local communities, and the need to retain local control of planning decisions. Some policy documents acknowledged the validity of this argument. Local hospitals were referred to as “…part of the community” and contributing “…to the local economy”.^28^ The association between hospitals and academic partners was advocated on the grounds that it would deliver “…financial gain and contribution to economic growth”,^28^ and “…realise for the people of [a given] region the economic and other benefits that flow from strong education and training, and leading edge health, research, technology and innovation”.^33^

Many external stakeholders and some internal stakeholders stressed the importance of retaining local control of hospital services. They spoke about the symbolic status of the hospital: *“…it's got to do with almost the town's worth and its identity and its self‐importance… If people are coming to develop, outside industrialists and investors, they'll ask is there an airport nearby, what's the road network, is there a university, ask if there's a hospital”* (Media, Region SE). Some internal stakeholders acknowledged the importance of hospitals to the local economy but were sceptical about the validity of this concern: *“People are horrified when you raise with them 'well actually it's not fit for purpose'. They don't care. It's there to employ people”* (Internal medical stakeholder, Region W/NW). This scepticism was particularly evident when internal stakeholders discussed the motivations of local politicians and their role in the planning process: *“It's all about votes”* (Internal medical stakeholder Region W/NW). Supporting the closure of a local Emergency Department was viewed as *“…political suicide”* (Internal medical stakeholder, Region W/NW). External stakeholders were also wary of the motives of politicians who took part in campaigns while in opposition but then abandoned their stance while in Government. They argued that sometimes planning decisions were influenced by the desire of local politicians to avoid being blamed by the public. This led to instances where a local service was *“…slowly strangle[d] to death”* through neglectful practices such as not filling empty consultant posts (Hospital campaigner, Region DS). This, they believed, minimised political damage by allowing hospitals to *“...wither on the vine”* where staffing was concerned (Hospital campaigner, Region DS). This kind of political interference was unanimously considered to be unjustified among internal and external stakeholders: *“…you've every local politician with a placard saying 'oh we want to keep this service in this hospital, our patients deserve it'. It's bloody daft, like you know, that needs to be dealt with"* (GP, Region D‐MID).

## 4 DISCUSSION

### 4.1 Summary of findings

This is a large‐scale qualitative study that presents a unique insight into the motivation for, and goals of, urgent and emergency care reconfiguration in the Republic of Ireland. While there was some variation in perspectives within the stakeholder cohorts, the internal and external stakeholder cohorts tended to use different lines of argument. Clinicians and management in the internal stakeholder group generally advocated arguments in favour of reconfiguration based on efficiency and safety claims. External stakeholders expressed arguments that focused on access to care and rebutted claims about efficiency and safety. The “voter” argument was mentioned by both internal and external stakeholders, often in a critical fashion. Overall, there was dissatisfaction with the state of public discourse in this field and with the overall quality of the policy making process.

Our findings broadly resonate with those of previous studies on health care system reconfiguration.^2^, ^10^ However, our study found a more unified position among internal clinical and managerial stakeholders than has previously been reported. Policy documents may have played a powerful role in framing centralisation as a clinical necessity, and many of these documents were influenced or written by senior clinical leaders in Ireland. This may have contributed to the co‐opting of internal clinical stakeholders to technical arguments in favour of reconfiguration and shaped their generally negative views on the arguments made by external stakeholders. The dominant influence of technical arguments in health planning is an emerging phenomenon in the literature on the discursive practice of policy.^15^, ^36^ Jones^15^ suggests that much current public discourse about health care delivery in its current form is influenced by a technocratic ideology which values abstract “expert” knowledge over and above the experiential “non‐expert” claims of the public and patients.

### 4.2 Efficiency arguments

Claims about the efficiency benefits of reconfiguration were disputed. Internal stakeholders argued that centralising urgent and emergency care would result in a more efficient system, but there was a marked scepticism about these claims among other interviewees. This scepticism is not unwarranted. Smith^37^ noted that conceptualising, measuring, and improving efficiency in health care is fraught with difficulty. A 2015 review by Imison et al^38^ of 123 proposed reconfiguration projects in the UK found no evidence of significant savings resulting from reconfiguration. It is noteworthy that most reconfiguration in Ireland occurred in rural areas, where significant investment in community and ambulance services would normally be required to alleviate the harms associated with reduced access.^10^ Reconfiguring services in urban areas particularly in Dublin, where 6 Emergency Departments continue to operate in close proximity to each other, would appear to offer greater scope for efficiency gains, without necessarily affecting the timeliness of access. It is unclear why major emergency care services in Dublin have not been centralised if efficiency is a genuine driver of reconfiguration in Ireland. It is possible that the 6 acute hospitals in Dublin are “protected” from rationalisation because of other factors which are not made explicit to the public when reconfiguration plans are discussed. These include the desirability of living and working in Dublin for many clinical staff compared with other parts of the country, and the importance of each Dublin hospital as training and research sites for the 3 medical schools in Dublin.

### 4.3 Patient safety arguments

Policy documents and internal stakeholder interviewees generally asserted the need to centralise complex emergency care at a small number of urban‐based hospitals. External stakeholders generally disagreed and argued that reconfiguration had increased the fragility of the care system in certain regions by relying on a single facility. They also argued that evidence about the volume‐outcome relationship only applies to a small proportion of the cases seen at Emergency Departments. While certainly relevant to discussions about the delineation of hospital services, the volume‐outcome relationship should not be the sole basis for policy‐making. Harrison^11^ cautions cherry picking or exaggerating the strength of the evidence because the volume‐outcome literature is not generalisable to every context and condition, and can result in high‐performing smaller units closing and poor‐performing larger units expanding. There is also evidence that centralisation of emergency care leads to increased travel time for patients living in rural areas,^39^, ^40^ and that distance to an Emergency Department is negatively associated with patient outcome.^40^, ^41^ Centralisation is not an “inevitable” interpretation of the literature in this field, and alternative approaches have been implemented in countries with challenging geographies. For example, certain Australian regions have developed policies and incentives to sustain local rural hospital facilities rather than transferring their resources and patients to larger centres.^42^

### 4.4 Access‐related arguments

Both policy documents and internal stakeholders tended to frame access and quality/safety as part of a larger “appropriateness” construct. The goal of reconfiguration was to design a system that delivered patients as quickly as possible to a facility that was appropriate for the severity of their condition. External stakeholders provided a nuanced critique of this vision highlighting the harms associated with increased travel times and concerns about the quality of care at the designated hub hospitals. It is notable that alternative models of care were given little or no consideration in either official policy documents or by internal stakeholders. The focus of policy makers was on ways to mitigate the harms associated with reduced access rather than how to avoid inducing these harms at all. It is also notable that complex care for rural populations in the North Eastern and Midlands regions is largely provided by hospitals in Dublin rather than by facilities within those regions. This has led to a situation where the major Dublin hospitals are now the de facto hubs for adjacent geographical regions despite being at the extreme periphery of those regions. This somewhat undermines the notion that access and quality/safety are given equal consideration when choices are made.

### 4.5 Arguments related to community values and local politics

External stakeholders argued for local control of services, stressing their role in supporting the broader health of community life. This argument has support in the international literature. Local hospitals have been found to play an important symbolic role in community life^43^ and are a major contributor to local economies.^44^, ^45^ Moreover, it has been suggested that downgrading the services provided by local hospitals may have a negative effect on perceptions about the overall quality of health care in a community.^46^ There was a notable degree of cynicism from both internal and external stakeholders regarding the motivations of politicians and their involvement in debates around health care. Nevertheless, some of the politicians we interviewed articulated the sense of value that communities placed in their local hospitals, offering an important counterbalance to the orthodox narrative of rationalisation. Jones^47^ notes that modern hospital planning is often driven by a coalition of managers and medical professionals and underpinned by an ideology of centralisation that does not necessarily align with the wishes of the public. This approach appears to value the views of specific interest groups over others within the plurality of perspectives around health care provision.^48^ It would be unfortunate if the cynical actions of some local politicians create a perception that any involvement by elected representatives in the planning of local health care services is “interference” rather than a natural part of the democratic process.

### 4.6 Limitations

There are some limitations to this study. The number of representatives interviewed from private emergency medicine, patient advocacy, and local media is disproportionately less than other stakeholder types. There is also a discrepancy in the total number of stakeholders interviewed in each region, with more in the South region than in other regions. However, the absolute number of interviewees in each stakeholder group was still relatively large by the normal standards used for qualitative research. It is also notable that the study findings were very consistent across regions, and it is unlikely that a larger sample size would have changed this.

### 4.7 Conclusions

This study adds to an emerging consensus that merely presenting communities with evidence to justify planned changes is not enough to persuade them to support reconfiguration^1^, ^48^ and may in fact contribute to public opposition by reinforcing the notion that “expert” opinion is more valuable than the views of the public.^48^ Arnstein^49^ has argued that information‐sharing approaches may be viewed as tokenistic or manipulative by the public, serving only to demonstrate the power imbalance between decision‐makers and citizens. A striking feature of our document analysis is the extent to which health planning in Ireland is led by health care providers such as the HSE, or by employees of the HSE acting in a consultative capacity. Ireland does not have independent health planning bodies, tasked with taking into account national and regional population needs and the views of patients and the public when considering the optimal configuration of services. This increases the danger that population health needs become secondary to the concerns of providers, such as difficulties with staffing smaller hospitals.

Our study portrays a confused and dysfunctional public discourse about urgent and emergency care provision in Ireland. Internal stakeholders are largely wedded to a “follow the evidence” model of decision making. This is despite concerns about the generalisability of the chosen evidence to different contexts in Ireland and the narrow focus on evidence about volume‐outcome relationships for a small number of conditions. External stakeholders present largely reactionary arguments, focused on rebutting the arguments put forward by those in charge of the reconfiguration process. The results of this study should be used to inform future health care reconfiguration initiatives in Ireland, both for urgent and emergency care, and for other sectors. Future consultation should be conducted in a way that genuinely respects the range of perspectives advanced by all stakeholders and not designed solely to overcome opposition.^50^ While it is unlikely that a “perfect” approach exists, there is evidence that certain factors are associated with positive public engagement. The best outcomes have been found in cases where the engagement process started at the early stages of planning, the public were offered opportunities for genuine interaction, the process was led by clinicians involved in delivering the service in question, and public representatives were engaged with.^50^ The need to move to pluralistic public engagement strategies is particularly pressing in an international policy landscape where authority and expertise are coming under increasing public scrutiny.

## Supporting information

Breakdown of policy documents analysed by domain
Topic guide for interviews

## References

[1] Fulop N , Walters R . Implementing changes to hospital services: factors influencing the process and ‘results’ of reconfiguration. Health Policy. 2012;104:128‐135. http://www.sciencedirect.com/science/article/pii/S0168851011001096 Accessed October 10, 2016 21719140 10.1016/j.healthpol.2011.05.015

[2] Farrington‐Douglas J , Brooks R . The future hospital: the politics of change. http://saluteinternazionale.info/wp-content/uploads/2009/04/future-hospital-2.pdf Accessed October 10, 2016.

[3] Bhattarai N , McMeekin P . Economic evaluations on centralisation of specialised healthcare services: a systematic review of methods. BMJ Open. 2016;6:1‐12. https://doi.org/10.1136/bmjopen-2016-011214 10.1136/bmjopen-2016-011214 PMC4861117 27154484

[4] Fosbol EL , Granger CB , Jollis JG , et al. The impact of a state wide pre‐hospital STEMI strategy to bypass hospitals without percutaneous coronary intervention capability on treatment times. Circulation. 2013;127:604‐612. https://doi.org/10.1161/CIRCULATIONAHA.112.118463 23275382 10.1161/CIRCULATIONAHA.112.118463

[5] Fulop N , Ramsay AG , Perry C , et al. Explaining outcomes in major system change: a qualitative study of implementing centralised acute stroke services in two large metropolitan regions in England. Implement Sci. 2016;11:80‐92. https://doi.org/10.1186/s13012-016-0445-z 27255558 10.1186/s13012-016-0445-z PMC4891887

[6] Metcalfe D , Bouamra O . Effect of regional trauma centralization on volume, injury severity and outcomes of injured patients admitted to trauma centres. Br J Surg. 2014;101(8):959‐964. https://doi.org/10.1002/bjs.9498 24915789 10.1002/bjs.9498

[7] Morris S , Hunter RM , Ramsay AG , et al. Impact of centralising acute stroke services in English metropolitan areas on mortality and length of hospital stay: difference‐in‐differences analysis. BMJ. 2014;349:g4757 https://doi.org/10.1136/bmj.g4757 25098169 10.1136/bmj.g4757 PMC4122734

[8] Barratt H , Raine R . Hospital service reconfiguration: the battle for hearts and minds. BMJ. 2012;344:20‐25. e980 10.1136/bmj.e953 22344306

[9] Barrett P . Safety, sustainability, accessibility—striking the right balance: reflections of a retiring Chair. London: Independent Reconfiguration Panel; 2012.

[10] Spurgeon P , Cooke M , Fulop N , et al. Evaluating models of service delivery: reconfiguration principles. http://www.netscc.ac.uk/hsdr/files/project/SDO_ES_08-1304-063_V01.pdf Accessed July 20, 2013.

[11] Harrison A . Assessing the relationship between volume and outcome in hospital services: implications for service centralisation. Health Serv Manage. 2012;25(1):1‐6. https://doi.org/10.1258/hsmr.2011.011027 10.1258/hsmr.2011.011027 22323665

[12] Lee JE , Sung JH . Utilization of the emergency room: impact of geographic distance. Geospat Health. 2007;1(2):243‐253. https://doi.org/10.4081/gh.2007.272 18686249 10.4081/gh.2007.272

[13] Turnbull J , Martin D . Does distance matter? Geographical variation in GP out‐of‐hours service use: an observational study. Br J Gen Pract. 2008;58(552):471‐477. https://doi.org/10.3399/bjgp08X319431 18611312 10.3399/bjgp08X319431 PMC2441507

[14] Nicholl J , West J . The relationship between distance to hospital and patient mortality in emergencies: an observational study. Emerg Med J. 2007;24(9):665‐668. https://doi.org/10.1136/emj.2007.047654 17711952 10.1136/emj.2007.047654 PMC2464671

[15] Jones L . Policy as discursive practice: an ethnographic study of hospital planning in England. PhD thesis 2016. London School of Hygiene & Tropical Medicine. https://researchonline.lshtm.ac.uk/2997234/1/2016_PHP_PhD_Jones_L.pdf Accessed April 25, 2017.

[16] Denis J , Langley A . Strategizing in pluralistic contexts: rethinking theoretical frames. Hum Relat. 2007;60:179‐215. https://doi.org/10.1177/0018726707075288 Accessed January 17, 2017

[17] Stewart E , Aitken M . Beyond NIMBYs and NOOMBYs: what can wind farm controversies teach us about public involvement in hospital closures? BMC Health Serv Res. 2015;15:530 https://doi.org/10.1186/s12913-015-1172-x 26626419 10.1186/s12913-015-1172-x PMC4667512

[18] 6 P . Hospital reconfiguration: issues from available recent literature. (Unpublished paper) Birmingham: Health Services Management Centre; 2004.

[19] Health Service Executive, Ireland . Transformation Programme 2007‐2010. http://www.hse.ie/eng/services/Publications/corporate/transformation.pdf Accessed July 5, 2013.

[20] Health Information and Quality Authority, Ireland . Report of the investigation into the quality and safety of services and supporting arrangements provided by the Health Service Executive at the Mid‐Western Regional Hospital Ennis, 2009 https://www.hiqa.ie/system/files/HIQA_Ennis_report_09042009.pdf Accessed September 10, 2013 .

[21] Health Information and Quality Authority, Ireland . Report of the investigation into the quality and safety of services and supporting arrangements provided by the Health Service Executive at Mallow General Hospital, 2011 https://www.hiqa.ie/system/files/Mallow_Guidance_Document.pdf Accessed September 10, 2013 .

[22] Central Statistics Office, Ireland . Summary results part 1. Table EY001: Population at each census from 1841 to 2016 by county, sex and census year. http://Statire/SelectVarVal/Statire/SelectVarVal/Define.asp?maintable=EY001&PLanguage=0 Accessed April 20, 2017.

[23] Dye TR . Understanding Public Policy. Prentice Hall: Englewood Cliffs, NJ; 1972.

[24] Bernier NF , Clavier C . Public health policy research: making the case for a political science approach. Health Promot Int. 2011;26(1):109‐115. https://doi.org/10.1093/heapro/daq079 21296911 10.1093/heapro/daq079

[25] Ritchie J , Lewis J . Qualitative research practice: a guide for social science students and researchers. London: Sage; 2003.

[26] Department of Health and Children, Ireland . Future health: a strategic framework for reform of the health service 2012 – 2015. http://health.gov.ie/wp-content/uploads/2014/03/Future_Health.pdf Accessed April 10, 2017.

[27] Department of Health and Children, Ireland . Securing the future of smaller hospitals: a framework for development. http://health.gov.ie/wp-content/uploads/2014/03/SecuringSmallerHospitals.pdf Accessed October 15, 2016.

[28] Department of Health and Children, Ireland . The establishment of hospital groups as a transition to independent hospital trusts. http://health.gov.ie/wp-content/uploads/2014/03/IndHospTrusts.pdf Accessed October 15, 2016.

[29] Health Service Executive, Ireland . National emergency medicine programme report: a strategy to improve safety, quality, access and value in emergency medicine in Ireland. http://www.hse.ie/eng/services/publications/Clinical-Strategy-and-Programmes/The-National-Emergency-Medicine-Programme.pdf Accessed July 5, 2013.

[30] Health Service Executive, Ireland . “Right Care, Right Now”. National clinical programme for critical care. http://hse.ie/eng/about/Who/clinical/natclinprog/criticalcareprogramme/modelofcare/criticalcare.pdf Accessed April 12, 2017.

[31] Royal College of Physicians of Ireland, Irish Association of Directors of Nursing and Midwifery . Report of the national acute medicine programme. http://www.hse.ie/eng/about/Who/clinical/natclinprog/acutemedicineprogramme/report.pdf Accessed July 5, 2013.

[32] Royal College of Physicians of Ireland and Health Service Executive . Model of care for acute surgery: national clinical programme in surgery. http://www.rcsi.ie/files/surgery/docs/20131030121710_RCSI_Model_of_Care_for_Acute_S.pdf Accessed July 9, 2013.

[33] Health Service Executive, South of Ireland . Reconfiguration of acute hospital services, Cork and Kerry: a roadmap to develop an integrated university hospital network. http://www.hse.ie/eng/services/News/Reconfiguration_Roadmap_Report_Cork_and_Kerry.pdf Accessed July 22, 2013.

[34] Horwath Consulting Ireland and Teamwork Management Services . Review of acute hospital services in HSE Mid‐West. An action plan for acute and community services, 2008a http://www.hse.ie/eng/services/publications/hospitals/Review_of_Acute_Hospital_Services_in_the_Mid-West.pdf Accessed August 1, 2013.

[35] Horwath Consulting Ireland and Teamwork Management Services . Review of acute services in HSE South and a five year action plan for Cork and Kerry, 2008b http://www.lenus.ie/hse/handle/10147/70013 Accessed August 1, 2013.

[36] Jones L , Exworthy M . Framing in policy processes: a case study from hospital planning in the National Health Service in England. Soc Sci Med. 2015;124:196‐204. https://doi.org/10.1016/j.socscimed.2014.11.046 25461877 10.1016/j.socscimed.2014.11.046

[37] Smith PC . What is the scope for health system efficiency gains and how can they be achieved? Eurohealth incorporating Euro Observer. 2012;18(3):1‐4. http://www.euro.who.int/__data/assets/pdf_file/0017/174410/EuroHealth-v18-n3.pdf Accessed April 20, 2017

[38] Imison C , Sonola L . Insights from the clinical assurance of service reconfiguration in the NHS: the drivers of reconfiguration and the evidence that underpins it—a mixed‐methods study. Health Serv Deliv Res. 2015;3(9). https://doi.org/10.3310/hsdr03090 25834885

[39] Matsumoto M , Ogawa T . The impact of rural hospital closures on equity of commuting time for haemodialysis patients: simulation analysis using the capacity‐distance model. Int J Health Geogr. 2012;11:28 https://doi.org/10.1186/1476-072X-11-28 22824294 10.1186/1476-072X-11-28 PMC3503736

[40] Shen Y‐C , Hsia RY . Does decreased access to emergency departments affect patient outcomes? Analysis of acute myocardial infarction population 1996‐2005. Health Serv Res. 2012;4(1 PART 1):188‐210. https://doi.org/10.1111/j.1475-6773.2011.01319.x 10.1111/j.1475-6773.2011.01319.x PMC3258371 22091922

[41] Hsia RY , Kanzaria HK , Srebotnjak T , Maselli J , McCulloch C , Auerbach AD . Is emergency department closure resulting in increased distance to the nearest emergency department associated with increased inpatient mortality. Ann Emerg Med. 2012;60(6):707‐715. https://doi.org/10.1016/j.annemergmed.2012.08.025 23026784 10.1016/j.annemergmed.2012.08.025 PMC4096136

[42] Rechel B , Dzakula A , Duran A , et al. Hospitals in rural or remote areas: an exploratory review of policies in 8 high‐income countries. Health Policy. 2016;120(7):758‐769. https://doi.org/10.1016/j.healthpol.2016.05.011 27312144 10.1016/j.healthpol.2016.05.011

[43] Imison C . Reconfiguring Hospital Services. London: Kings Fund; 2011.

[44] Doeksen GA , Johnson T . Measuring the economic importance of the health sector on a local economy: a brief literature review and procedures to measure local impacts. Mississippi State 39762: Mississippi State University, Southern Rural Development Center; 1997 http://srdc.msstate.edu/publications/archive/202.pdf Accessed March 1 2017.

[45] McDermott RE , Cornia GC . The economic impact of hospitals on rural communities. J Rural Health. 1991;7(2):117‐133. https://doi.org/10.1111/j.1748-0361.1991.tb00714.x 10116773 10.1111/j.1748-0361.1991.tb00714.x

[46] Carson S , Peterson K . Evidence Brief: Effects of Small Hospital Closure on Patient Health Outcomes. NCBI Resources. Department of Veterans Affairs, Veterans Health Administration, Quality Enhancement Research Initiative, Washington DC: 2013. 27606393

[47] Jones L . What does a hospital mean? J Health Serv Res Policy. 2015;20(4):254‐256. https://doi.org/10.1177/1355819615585893 26041680 10.1177/1355819615585893

[48] Barratt H , Harrison DA . Factors that influence the way local communities respond to consultation processes about major service change: a qualitative study. Health Policy. 2015;119(9):1210‐1217. https://doi.org/10.1016/j.healthpol.2015.04.015 25975768 10.1016/j.healthpol.2015.04.015 PMC4561526

[49] Arnstein SR . A ladder of citizen participation. J Am Plann Assoc. 1969;35(4):216‐224. https://doi.org/10.1080/01944366908977225

[50] Dalton J , Chambers D . Service user engagement and health service reconfiguration: a rapid evidence synthesis. Health Serv Deliv Res 2015; 3 17 https://www.ncbi.nlm.nih.gov/pubmed/25927128. Accessed January 10, 2017. 25927128

