# Supplementary material for: Perspectives on the underlying drivers of urgent and emergency care reconfiguration in Ireland
Source: Int J Health Plann Manage. 2017 Oct 26;33(2):364–79. doi: 10.1002/hpm.2469 (PMC6032929; doi:10.1002/hpm.2469)
Supplement: Supplementary file 1 — Breakdown of policy documents analysed by domain Topic guide for interviews [file HPM-33-364-s001.docx]

| **TOPIC GUIDE** | |
| --- | --- |
| **Background Questions** | **Prompts/Probes** |
| 1. **Can you tell me a little about your role and how you are connected to urgent & emergency care in this region/in Ireland?** | - Nature (operational/strategic) & length? |
| 1. **What is your understanding of urgent & emergency care?** | ***-*** What does it involve? |
| 1. **How is this care currently delivered in your local area (rural/urban)?**  - **Geography** - **Model** | ***Geography***  - Boundaries/catchment areas?  ***Model***  - Your local GP co-op service?  ***-*** Your local hospital’s service?  - Relationship between your local hospital & other hospitals in this region?  - Relationships between the hospitals, primary care (including GP co-ops), CITs & ambulance service?  - Governance structures to support this network? Clinical and corporate decision-making?  - Culture? Staff morale? Staff turnover? Patient satisfaction? |
| 1. **What are your thoughts on how this care is delivered in your local area (rural/urban)?**   *Appropriate care in the appropriate setting. Right place, right time.*   - **Strengths** - **Weaknesses** - **Solutions?** | - Strengths & weaknesses of the system of care pathways (primary, community intervention, ambulance & hospital?  **Access?**  **.1.** Ease of access **ie.** GP co-op, Public/private mix, distance, ambulance service  Capacity (include RRVs & ICVs), priority setting & off-load time, ED waiting time, & ED + in-patient capacity  **.2.** Appropriate referral **ie.** Self/GP/inter-professional?  **Cost? Is this system cost effective? Everyone might be doing their best but should we be looking at a more efficient way of doing things?**  Rates of attendance/leaving before completion of treatment/re-attendance within 7 days/admission/re-admission, as well as length of stay & resources?  **Quality & Safety? Is this provision of care safe? Of high quality? How would a system approach improve this care?**  Staffing, skilling up/down, role expansion, supervision & cover, rotation, turnover, patient safety, satisfaction & dignity? |
| 1. Are current practices sustainable? | - Compliance/divergence? Is there a need for change or not? Why? |
| **Questions on planning change** | **Prompts/Probes** |
| 1. **Has it always been like that?**  - **Change** - **Timeline** | ***Explore the history of this care arrangement in terms of past/present/future plans***  - Model (infrastructure, hospital & system relationships & governance structures)? |
| 1. **Were there any events that triggered plans for change/called for a need for change in how this care should be delivered?**  - **Evidence** | - Examples & impact?  Eg: Teamwork management services reports, HIQA reports on quality, safety & governance, international evidence, Primary Care Strategy, draft National Ambulance Plan, AMP, & NEMP? |
| 1. **What are your thoughts on what drove/is driving the need for change?**  - **Goals** | - **Consumer:** Ease of access & convenience?  - **Economic:** Cost & resource efficiency (staffing, supervision, cover)?  - **Patient:** Quality & safety improvements (stroke incidence, heart failure, diabetes & consequences)?  - **Political:** Community employment & development? Political accountability to jurisdiction? |
| 1. **Who was/is/should be involved in planning change?**  - **Names** - **Roles** | ***Who consulted with whom AND who did you consult with?***  - Internal stakeholders & role (informal/formal)?  - External stakeholders & role (informal/formal)?  **- Your involvement?** |
| 1. **What do you think about how the general public & clinical professionals were/are/should be involved in planning change?**  - **Issues** - **Trade-offs** - **Influence** | - Specific issues for discussion?  - Frequency & informal/formal nature of discussion?  - Attempts to illicit trade-offs between access, quality (structure & resources) & cost?  - Influence on final models chosen?  **- Your involvement?** |
| 1. **Are there plans for change that have yet to be implemented?**  - **Changes to the original plan** - **6 hospital group structure** - **Timelines** | - Changes to the original plan – Why? Have some changes been implemented already? **Were they independent of reconfiguration** (eg. cost-cutting, expanded group control)**?**  - 6 hospital group structure – Model (infrastructure, hospital & system relationships & governance structures)?  - Timelines? |
| **Questions on impact of change** | **Prompts/Probes** |
| 1. **What has been/will be the impact of these changes in your local area (rural/urban)?** | - Strengths & weaknesses of the system of care pathways – **Access, Cost, Quality & Safety**? |
| 1. **How sustainable are/will these changes (be)?** | - What practices (will) work or otherwise? Why?  - Is/will there be a need to revert back to the former practices or propose further changes? |
| 1. **Is (will) your local hospital/network currentlyevaluating the quality of care being provided, in light of those changes (that are to come about)?** | ***-*** Examples of quality dimensions?  - Suggestions of measures useful to evaluate this system of care & inform improvements?  - **Suggest 1 measure that you would like to see collected?** |
| 1. **Do you see a role for academia in the provision of urgent & emergency care going forward?** | - Examples of benefits include hospital viability, teaching, training, research, innovation & commercialisation? |
| 1. **What would you consider to be the ideal system of urgent & emergency care?** | - Goals? Infrastructure? Evaluation techniques? |
| 1. **What would be your advice to another team about to embark on a similar change in this area of care?** |  |
| 1. **Are there other relevant issues relating to the delivery of urgent & emergency care that have not yet been raised in this interview?** |  |
| 1. **Are there any relevant documents that have been published about the reconfiguration of EUCS in this are?** |  |
| 1. **Can you suggest other key stakeholders (internal or external) that we should contact?** |  |

| **BREAKDOWN OF POLICY DOCUMENTS ANALYSED BY DOMAIN** | |
| --- | --- |
| **.1.** | **Policy documents relating to all or part of the provision of urgent and emergency care**  College of Psychiatrists of Ireland, 2006. A vision for change. Report of the expert group on mental health policy. Department of Health and Children, Dublin.  Department of Health and Children, 2008. Building a Culture of Patient Safety: Report of the Commission on Patient Safety and Quality Assurance. Department of Health and Children, Dublin.  Department of Health and Children, 2012. Future Health: A Strategic Framework for Reform of the Health Service 2012 – 2015. Department of Health and Children, Dublin.  Health Service Executive, 2010. National Review of GP Out of Hours Services. Health Service Executive, Kildare.  Health Service Executive, 2013. National Ambulance Service Plan. Health Service Executive, Kildare. |
| **.2.** | **National hospital reconfiguration reports**  Department of Health and Children, 2013a. The Establishment of Hospital Groups as a Transition to Independent Hospital Trusts. Department of Health and Children, Dublin.  Department of Health and Children, 2013b. Securing the Future of Smaller Hospitals: A Framework for Development. Department of Health and Children, Dublin.  Health Service Executive, 2006. Transformation Programme 2007-2010. Health Service Executive, Kildare. |
| **.3.** | **Regional hospital reconfiguration reports**  Health Service Executive, South, 2010. Reconfiguration of acute hospital services, Cork and Kerry: A roadmap to develop an integrated university hospital network. Health Service Executive, South, Cork.  Horwath Consulting Ireland and Teamwork Management Services, 2008a. Review of Acute Hospital Services in HSE Mid-West. An action plan for acute and community services. Health Service Executive, Kildare.  Horwath Consulting Ireland and Teamwork Management Services, 2008b. Review of acute services in HSE South and a five year action plan for Cork and Kerry. Health Service Executive, Kildare.  Teamwork Management Services, 2006. Improving safety and achieving better standards. An Action Plan for Health Services in the North East. Health Service Executive North-East, Navan. |
| **.4.** | **Healthcare regulatory reports at specific hospital sites**  Health Information and Quality Authority, 2009. Report of the investigation into the quality and safety of services and supporting arrangements provided by the Health Service Executive at the Mid-Western Regional Hospital Ennis. Health Information and Quality Authority, Cork.  Health Information and Quality Authority, 2011. Report of the investigation into the quality and safety of services and supporting arrangements provided by the Health Service Executive at Mallow General Hospital. Health Information and Quality Authority, Cork.  Health Information and Quality Authority, 2012. Report of the investigation into the quality and safety of services and supporting arrangements provided by the Adelaide and Meath Hospital, Dublin incorporating the National Children’s Hospital (AMNCH) for patients who require acute admission. Health Information and Quality Authority, Cork.  Health Information and Quality Authority, 2014. Review of the governance arrangements as reflected in the safety, quality and standards of services at University Limerick Hospitals. Health Information and Quality Authority, Cork.  Health Information and Quality Authority, 2015. Report on the Investigation into the safety, quality and standards of services provided by the Health Service Executive to patients in the Midland Regional Hospital Portlaoise. Health Information and Quality Authority, Cork. |
| **.5.** | **National clinical programme reports**  Department of Health and Children, 2006. Strategy for Cancer Control in Ireland. Department of Health and Children, Dublin.  Health Service Executive, 2012. National emergency medicine programme report: A strategy to improve safety, quality, access and value in emergency medicine in Ireland. Health Service Executive, Kildare.  Health Service Executive, 2014. “Right Care, Right Now”. National clinical programme for critical care. Health Service Executive, Kildare  Royal College of Physicians of Ireland et al., 2010. Report of the national acute medicine programme. Health Service Executive, Kildare.  Royal College of Physicians of Ireland and Health Service Executive, 2013. Model of Care for Acute Surgery: National Clinical Programme in Surgery. Health Service Executive, Kildare. |
